# Airborne Particulate Matter in School Classrooms of Northern Italy

**DOI:** 10.3390/ijerph110201398

**Published:** 2014-01-27

**Authors:** Sabrina Rovelli, Andrea Cattaneo, Camilla P. Nuzzi, Andrea Spinazzè, Silvia Piazza, Paolo Carrer, Domenico M. Cavallo

**Affiliations:** 1 Dipartimento di Scienza e Alta Tecnologia, Università degli Studi dell’Insubria, via Vallegio 11, Como 22100, Italy; E-Mails: andrea.cattaneo@uninsubria.it (A.C.); camilla.nuzzi@uninsubria.it (C.P.N.); andrea.spinazze@uninsubria.it (A.S.); domenico.cavallo@uninsubria.it (D.M.C.); 2 Dipartimento di Scienze Biomediche e Cliniche “L. Sacco”, Università degli Studi di Milano, via G.B. Grassi 74, Milano 20157, Italy; E-Mails: silvia.piazza@unimi.it (S.P.); paolo.carrer@unimi.it (P.C.)

**Keywords:** school environment, indoor PM sources, PM exposure assessment, exposure mitigation, intervention measures, risk management

## Abstract

Indoor size-fractioned particulate matter (PM) was measured in seven schools in Milan, to characterize their concentration levels in classrooms, compare the measured concentrations with the recommended guideline values, and provide a preliminary assessment of the efficacy of the intervention measures, based on the guidelines developed by the Italian Ministry of Healthand applied to mitigate exposure to undesirable air pollutants. Indoor sampling was performed from Monday morning to Friday afternoon in three classrooms of each school and was repeated in winter 2011–2012 and 2012–2013. Simultaneously, PM_2.5 _samples were also collected outdoors. Two different photometers were used to collect the PM continuous data, which were corrected *a posteriori* using simultaneous gravimetric PM_2.5_ measurements. Furthermore, the concentrations of carbon dioxide (CO_2_) were monitored and used to determine the Air Exchange Rates in the classrooms. The results revealed poor IAQ in the school environment. In several cases, the PM_2.5_ and PM_10_ 24 h concentrations exceeded the 24 h guideline values established by the World Health Organization (WHO). In addition, the indoor CO_2_ levels often surpassed the CO_2_ ASHRAE Standard. Our findings confirmed that important indoor sources (human movements, personal clouds, cleaning activities) emitted coarse particles, markedly increasing the measured PM during school hours. In general, the mean PM_2.5_ indoor concentrations were lower than the average outdoor PM_2.5_ levels, with I/O ratios generally <1. Fine PM was less affected by indoor sources, exerting a major impact on the PM_1__–2.5_ fraction. Over half of the indoor fine particles were estimated to originate from outdoors. To a first approximation, the intervention proposed to reduce indoor particle levels did not seem to significantly influence the indoor fine PM concentrations. Conversely, the frequent opening of doors and windows appeared to significantly contribute to the reduction of the average indoor CO_2_ levels.

## 1. Introduction

The inhabitants of industrialized countries spend more than 90% of their time in indoor environments, where some air pollutants can reach levels far greater than those found outdoors [1]. As a consequence, in recent years, the Indoor Air Quality (IAQ) of residential buildings and workplaces has become an increasing interest for scientists and the public. Moreover, specific guidelines were recently developed by the World Health Organization (WHO) [2].

Indoor air pollution is of significant concern due to the numerous potential health hazards it presents to a large fraction of the population, including susceptible groups such as children, the elderly or people affected by chronic diseases [3]. Therefore, a good IAQ in homes, offices, and schools is crucial to ensure public wellbeing, as the presence of various indoor stressors can lead to short-term and long-term adverse effects [4]. 

Indoor air pollution is the result of physical, chemical and biological factors that are often present simultaneously and is determined by the local outdoor air, specific building characteristics and furnishings, ventilation systems, and human and indoor activities [5,6].

Schools in particular represent an important microenvironment, and the promotion of good IAQ in schools is of special relevance because the majority of children in Europe spend at least 30% of their week-days inside school buildings; furthermore, children are exposed to greater amounts of air pollution because they breath higher volumes of air in comparison with adults due to their low body weight and developing immune systems [7].

The importance of IAQ in school environments has been underlined by a large number of studies worldwide [5,6,8,9]. Poor IAQ in classrooms can increase the possibility of health problems for occupants, in addition to reducing scholarly performance, the attendance of students and ambient comfort [9], whereas good air quality can enhance children’s concentration as well as teacher productivity [10].

Particulate matter (PM) is one of the most common pollutants that could potentially reduce air quality in classrooms. Indoor PM levels derive from both indoor and outdoor sources and are influenced by numerous variables, such as air exchange rates and infiltration processes, outdoor air pollution levels, the type and intensity of indoor activities, and the aerodynamic diameters of the particles [11]. Although in school buildings many principal indoor PM sources, such as smoking and cooking, are not usually present, several studies have demonstrated that exposure to airborne particles in classrooms can nonetheless be high [11,12,13,14]. This may be due to: (a) insufficient and inadequate ventilation, especially in winter; (b) infrequently cleaned indoor surfaces; and (c) a large number of students in relation to the room area and volume, whose movements cause the re-suspension of settled particles. Some authors have determined that re-suspension is a significant factor affecting indoor particle concentrations, with suspension rates increasing with particle size; this phenomenon is particularly relevant for particles >1 µm and is even more important for particles in the 5–10 µm range [15]. The presence of children and their movements can also affect indoor PM levels through the interception of personal clouds (primarily comprising coarse particles) recorded by the PM sampling devices. Fromme *et al.* found an average indoor PM_2.5_ concentration higher than the corresponding outdoor level in a German primary school [12]. Similar findings have been confirmed by a Belgian survey [16]. Oeder *et al.* reported indoor PM_10 _concentrations 5 times higher than outdoor levels for six schools in Munich [17]. 

In Italy a prevention program for indoor environments is provided in the guidelines developed by the Ministry of Health “*Schema di linee di indirizzo per la prevenzione nelle scuole dei fattori di rischio indoor per allergie e asma* (Italian Ministry of Health, 2010) [18]. In this context, the Ministry of Health has promoted the “Indoor school” project (CCM 2010) whose main objective was the implementation of these guidelines.

The first phase of the project involved 29 public schools of Milan and assessed the knowledge of the school principals on issues related to IAQ and building characteristics of the schools through a questionnaire. In the second phase seven schools were selected, considering the year of construction, the characteristics of the schools and the traffic exposure, to assess the indoor environment by measurement of physical characteristics, chemical pollutants and biological agents and to assess its impact on health through questionnaires and clinical tests.

The primary aims of the present paper were to investigate the PM mass concentrations in elementary and secondary schools in Milan to evaluate the impact of indoor activities or outdoor sources on these levels, and to compare the measured indoor concentrations with recommended IAQ values. 

Furthermore, the efficacy of some intervention measures promoted to improve IAQ in school environments was assessed. Indeed, to date, only a limited number of surveys have tackled the problem of assessing PM exposure following risk mitigation interventions. Thus, a preliminary analysis of the measures undertaken to reduce the exposure of school occupants to airborne pollutants will be proposed. 

## 2. Materials and Methods

### 2.1. Study Design

Airborne particulate samples (as Total Suspended Particulates (TSP), PM_10_, PM_5_, PM_2.5_, PM_1_, PM_0.5_ and particles in the size ranges 0.3–0.5–1–2.5–5–10–>10 μm) were obtained from seven selected schools (three primary schools and four secondary schools) in the urban area of Milan, a large metropolitan city of 1,345,000 inhabitants located in the center of the Po Valley, which is the most industrialized and populated area of Italy.

**Figure 1:**
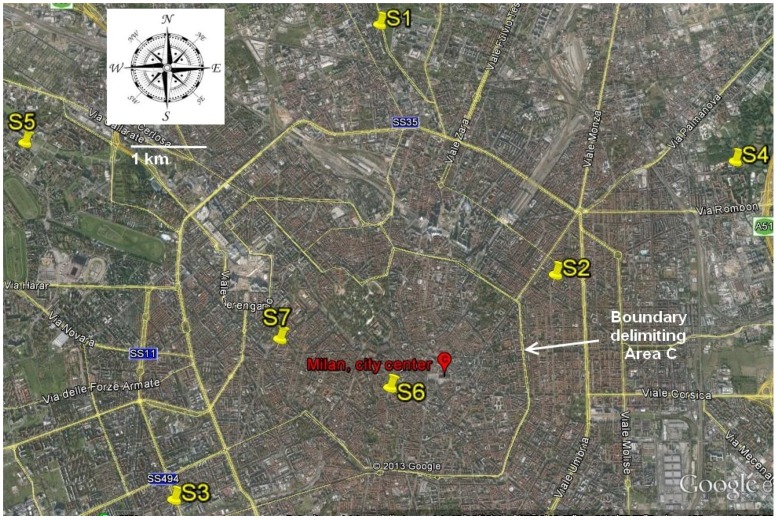
Location of study schools (S1 to S7).

As depicted in Figure 1, the school buildings identified with the initials S1 to S7 were distributed around the city center. S6 was located within the so-called “Area C” a limited traffic zone located in the center of Milan that prohibits the circulation of diesel cars Euro 0, 1, 2 and 3, while a ticket is required for vehicles meeting the other Euro standards. Schools S2 and S7 were just outside this area and the other four schools were located in suburban areas at a maximum distance of 7 kilometers from the center. More details are reported in Table 1. 

The selected schools were investigated during two sampling campaigns; the first (SC1) from November 2011 to March 2012 and the second (SC2) during the following winter, from November 2012 to February 2013. School S6 was monitored only during winter 2011–2012. Moreover, prior to the start of SC2, intervention measures were proposed in four schools (S1, S2, S4 and S5) to improve the IAQ in classrooms, through meetings with school principals, teachers and pupils, by the implementation of the guidelines developed by the Italian Ministry of Health (Italian Ministry of Health, 2010) [18]. The measures suggested daily cleaning of all classroom surfaces (blackboards, desks, chairs) with damp floor cloths, the cleaning of floors with vacuum cleaners equipped with High Efficiency Particulate Air Filters (HEPA), and ensuring adequate ventilation rates through the frequent opening of doors and windows. A limitation of the study is that no information about time-activity patterns were available, thus making the interpretation of results somewhat uncertain.

In every building, the measurements were conducted once for each monitoring session over five consecutive days during the school week, from Monday morning (8/8:30 AM) to Friday afternoon (4/4:30 PM), following a detailed operating protocol. A pilot study lasting two days was conducted prior to the start of sampling to evaluate the sampling protocols for potential sources of error.

**Figure 2:**
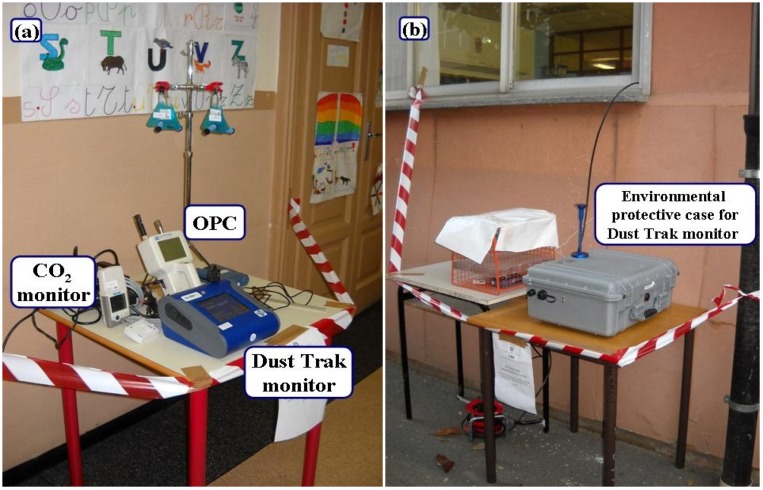
The indoor (**a**) and outdoor (**b**) sampling site. The PM and CO_2_ monitors are showed.

Within each school, the PM_2.5_ indoor samples were collected in the same three classrooms during both sampling sessions. Furthermore, in some of the investigated rooms (8 classrooms during SC1 and 12 during SC2), airborne particles within 6 size intervals ranging from 0.3 µm to particles >10 µm were also monitored. All the rooms were naturally ventilated and were provided with at least one blackboard with chalk. Table 1 summarizes some information about the classrooms where the measurements have been performed. Sampling devices were placed at the same sampling site in classrooms, away from blackboards, at a distance of over 50 cm from walls and approximately 1 m above the ground, which corresponded to the breathing zone of the occupants. Simultaneously, in each school, PM_2.5_ air samples were also collected outdoors following the same sampling protocol but using environmental protective cases to shield the instruments against rainfall (Figure 2).

Furthermore, the indoor and outdoor concentrations of carbon dioxide (CO_2_) were collected and used to determine the Air Exchange Rates (AERs) in classrooms. AERs were then employed to estimate the outdoor air supply rates per person (Q_p_) using the class volumes and the average number of students occupying the rooms [19]. 

All the weekly concentrations are referred to an average exposure time of 5-days (5-d), from Monday morning to Friday afternoon. 

### 2.2. Sampling Methods

#### 2.2.1. PM

Continuous measurements of indoor and outdoor PM_2.5_ with a sampling interval of 1 min were performed using a nephelometer (DustTrak ^TM^ II Aerosol Monitor Model 8530, TSI Inc., Shoreview, MN, USA) operating at 3 L/min and equipped with a size-selective impactor for PM_2.5 _(Figure 2). 

**Table 1:** Specific school and room characteristics, number of occupants and average occupancy of classrooms; e.s. = elementary school, s.s. = secondary school, s.n.d. = sampling not done.

School	Type of Site	Room	Classroom Grade—Age of Children	Floor and Direction of Classrooms	Floor Area (m^2^)	Room Volume (m^3^)	Number of Students ^a^		Occupancy (N Person/100 m^2^)	
							SC1	SC2	SC1	SC2
S1 (s.s.)	Suburban, at about 100 m from a main road (Viale Enrico Fermi)	R1	Second grade—12y (SC1); third grade—13y (SC2)	First floor, on the schoolyard	46.6	140.0	22.6	25.0	48	54
		R2			46.8	140.5	25.0	24.0	53	51
		R3			48.0	144.0	24.2	25.0	50	52
S2 (e.s.)	Urban (just outside Area C)	R1	Third grade—8y (SC1); fourth grade —9y (SC2)	Second floor, on the schoolyard	61.7	271.4	17.6	19.0	29	31
		R2			64.0	281.4	18.2	17.6	28	28
		R3			64.0	281.4	17.6	19.0	28	30
S3 (s.s.)	Suburban	R1	First grade—11y (SC1); second grade —12y (SC2)	Ground floor, on the schoolyard	49.9	149.6	22.4	22.2	45	44
		R2	Second grade—12y (SC1); third grade—13y (SC2)		46.2	143.0	24.6	23.8	53	52
		R3	First grade—11y (SC1); second grade —12y (SC2)	Ground floor, on a private street	58.9	176.8	22.8	20.6	39	35
S4 (e.s.)	Suburban, at about 1 Km from a highway (A51)	R1	Fourth grade—9y (SC1); fifth grade—10y (SC2)	Ground floor, on the schoolyard	49.7	159.0	22.2	20.8	45	42
		R2			49.7	159.0	20.8	23.0	42	46
		R3			49.7	159.0	20.2	22.2	41	45
S5 (s.s.)	Suburban	R1	First grade—11y (SC1); second grade—12y (SC2)	First floor, on the schoolyard	41.4	132.5	22.8	20.6	55	50
		R2		Second floor, on the schoolyard	43.2	138.0	21.6	20.6	50	48
		R3		Third floor, on the school yard	44.2	141.5	16.8	19.2	38	43
S6 (s.s.)	Urban (in Area C)	R1	Second grade—12y (SC1)	Second floor, on the schoolyard	33.0	108.9	18.0		55	
		R2			54.7	180.0	24.6	s.n.d.	45	s.n.d.
		R3			35.8	118.0	26.6		74	
S7 (e.s.)	Urban (just outside Area C)	R1	Fourth grade—9y (SC1); first grade—6y (SC2)	First floor, on the schoolyard	57.9	254.5	15.8	21.2	27	37
		R2	Fourth grade—9y (SC1); fifht grade—10y (SC2)	Second floor, on the schoolyard	48.0	192.0	24.0	24.8	50	52
		R3			48.0	192.0	19.0	20.4	40	43

Note:^ a^ Average number of students occupying the classrooms during the sampling week.

After each sampling, the impactors were cleaned and greased, the instrument was zeroed following the manufacturer’s instructions, and a pre- and post- calibration of the flow rate was conducted using a primary standard bubble-meter (Mini-Buck Calibrator M30, Buck Co., Orlando, FL, USA) to improve the accuracy of the sampling volume estimates and verify that considerable flux variations did not occur during sampling (<5%). 

The collection of PM_2.5_ on internal filters (PTFE membranes with a 2 µm pore size and 37 mm diameter) was conducted in every sampling session to assess the accuracy of the instrument and to correct the data *a posteriori*. Indeed, the factory calibration factor cannot be used to obtain accurate data when the analyzed particulates exhibit different morphologies, size distributions and chemical compositions as well as densities that differ significantly from the standard ISO 12103-1, A1 test dust. Because urban particulates in Milan are typically less dense than standard dust [20], a significant overestimation of concentrations is possible. This hazard has been addressed in the literature, which notes that PM levels measured by photometers may be 2-5 times greater than the reference gravimetrical method and that the differences increase when the ultrafine fraction predominates [21]. 

A photometer (OPC, Lighthouse 3016-IAQ, Fremont, CA, USA) was also used to collect continuous measurements of size-fractioned PM (PM_0.5_, PM_1_, PM_2.5_, PM_5_, PM_10_ and TSP) and airborne particles within the size ranges 0.3–0.5–1–2.5–5–10–>10 µm (Figure 2).

The PM_2.5_ mass concentrations determined by the OPC with a sampling interval of 2.5 minutes were corrected *a posteriori* with the PM_2.5_ gravimetric data obtained using the nephelometer. The same correction factor was also used to correct the PM optical data assuming the same density for PM_2.5 _and all other PM fractions. Thus, the mass concentrations of the other PM classes are not expected to be as accurate as those of PM_2.5_. If gravimetric sampling in the same room was not possible, the average gravimetric data collected in the other two classrooms of the same school was used for the correction.

#### 2.2.2. CO_2_

Indoor CO_2_ levels were monitored with a high sampling frequency (2.5 min) using a non-dispersive infrared analyzer (GE sensing Telaire 7001, Goleta, CA, USA) with a battery-operated data logger (Hobo H8/U12; Onset Computer Inc., Pocasset, MA, USA) (Figure 2) to assess the short-term variations and differences between school hours and other periods.

Before and after each sampling, all the monitors were set to zero through the flux of N_2 _at 0.5 bar, and the calibration was verified in a glove bag with a span gas bottle (1,000 ppm). Data were corrected *a posteriori* only when the accuracy exceeded the ± 10% range. 

### 2.3. Analytical Procedures

The mass of PM_2.5_ collected on membrane filters was determined gravimetrically following the standard operating procedure. Before weighing, each filter was conditioned at 50% ± 5% relative humidity and 20 °C ± 1 °C for a minimum of 24 hours in a climatic cabinet (Activa Climatic Cabinet, Acquaria; Lacchiarella, Milan, Italy). The filters were weighed three times (every 20′′) by a micro-balance with a precision of 1 µg (Mettler M3, W. Pabisch spa, Milan, Italy), ensuring a standard deviation ≤ 3 µg. An electrical C-shaped ionizer (HAUG GmbH & CO. KG, Osnabruck, Germany) was used to eliminate electrostatic charges from the filter surfaces. This procedure was repeated before and after each sampling, and the mass of the PM was determined by differential weighing. Two laboratory blanks were always weighed under the same conditions to verify possible anomalies during the conditioning. Prior to the analysis, the micro-balance was auto-calibrated, and the calibration check was performed using certified standard weights of 1 and 100 mg, allowing deviations from the true value ≤ 3 µg.

### 2.4. Data Analysis

The average indoor-generated PM_2.5_ concentration (C_ig_) was estimated though a regression analysis of the indoor (C_i_) *versus* outdoor (C_o_) gravimetrical concentrations. We chose to use the gravimetrical data collected by the nephelometers due to their greater accuracy relative to the optical data. Our model was based on the following Equation [22]:

C_i_ = C_og_ + C_ig_ = F_i_C_o_ + C_ig_ (1)

The slope of the regression estimates the infiltration factor (Fi), while the intercept estimates the C_ig_. Furthermore, the outdoor-generated average value (C_og_) was estimated as the difference between the total indoor PM_2.5_ level and the corresponding fraction of C_ig_. 

Indoor-outdoor AERs were estimated using the tracer gas decay technique employing occupant-generated CO_2_ as a tracer gas to estimate the ventilation rate after occupants left the building [19]. Specifically, the decay rate of metabolic CO_2 _was observed at the end of lessons, when no occupants were present in the rooms and the windows and doors were closed (typically after 5:30/6:00 PM). The AERs were estimated for each classroom when a good exponential decay curve was observed.

Descriptive statistics and other statistical analysis parameters were determined using the SPSS package (SPSS Inc., Chicago, IL, USA). The Gaussian distribution of the variables was verified by the Kolmogorov-Smirnov test. The correlations between the variables were expressed by Spearman correlation coefficients because the continuous data distribution was neither normal nor log-normal.

The differences between the average values collected during different time periods and between the 5-d average pre- and post-intervention concentrations were analyzed by the paired *t-*test, as the data sets exhibited a normal distribution.

## 3. Results

Table 1 summarizes the specific room characteristics and the average number of people occupying the classrooms during the sampling weeks. The occupancy of every monitored classroom, in terms of number of people per 100 m^2^, was calculated. 

The weather conditions—rainfall, relative humidity and wind speed—collected by the fixed monitoring stations of the Regional Agency for Prevention and Environment of Lombardy in the same weeks over which the indoor measurements were performed are outlined in Table 2. 

Descriptive statistics for the 5-d values of all the PM fractions collected during both sampling campaigns and throughout the survey are provided in Table 3. The indoor and outdoor CO_2_ values are also reported. During SC1, 1.9% of the indoor continuous CO_2_ data were underestimated due to the use of data loggers with a maximum recordable concentration of 2,485 ppm.

### 3.1. Indoor and Outdoor PM_2.5_ Levels

In general, the 5-d mean indoor PM_2.5_ concentration measured in the school environments was lower than the average outdoor PM_2.5_ level (Table 3). This trend was observed in all of the schools, with the exception of school S1 during the first sampling campaign, and, to a lesser extent, schools S2 and S4 during the second campaign, in which the 5-d indoor PM_2.5_ levels were higher (33.8 µg/m^3^, 13.5 µg/m^3 ^and 31.6 µg/m^3^, respectively) than the associated outdoor mean concentrations (23.1 µg/m^3^, 12.0 µg/m^3 ^and 27.3 µg/m^3^, respectively). 

The ratios between the 5-d indoor and outdoor PM_2.5_ levels were calculated for every monitored classroom and ranged from 0.48 to 1.50 (mean = 0.83, median = 0.73).

**Table 2:** Summary of the 5-d weather conditions during the experimental campaigns; n.a. = not available, s.n.d. = sampling not done.

	Rainfall (mm) ^a^		% RH		Wind Speed (m/s)	
School	SC1	SC2	SC1	SC2	SC1	SC2
S1	17.6	0	88.0	78.1	1.5	1.0
S2	0	34.4	78.6	84.6	0.9	2.2
S3	5.4	n.a.	92.7	55.5	1.2	1.1
S4	0	5.8	86.9	84.6	1.2	1.3
S5	0	0	64.2	88.8	1.3	1.3
S6	0	s.n.d.	55.3	s.n.d.	1.6	s.n.d.
S7	0	6	49.8	75.3	1.3	1.7
Total	23	46.2	73.6	77.8	1.3	1.4

Note: ^a^ mm of cumulative rainfall during every sampling week.

**Figure 3:**
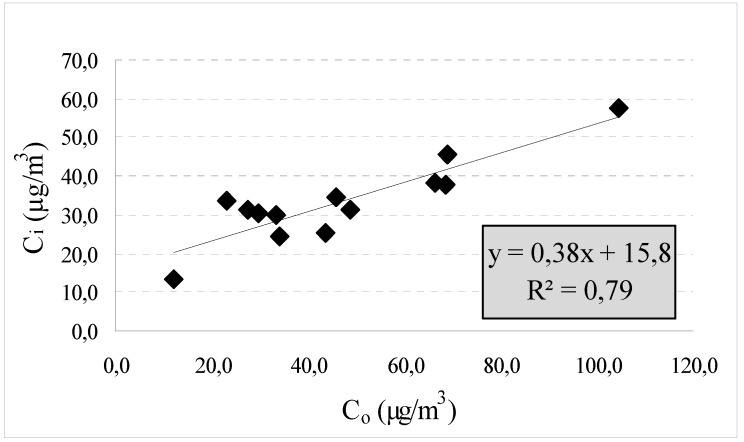
Linear regression between the 5-d gravimetrical outdoor PM_2.5_ concentrations (C_o_) and the corresponding indoor levels (C_i_) for every school building during both monitoring sessions.

The indoor and outdoor levels showed quite good correlations, as the Spearman’s coefficients between the indoor optical data and the corresponding outdoor concentrations for each classroom during both sampling campaigns were <0.5 for 4 out of the 37 classrooms and between 0.54 and 0.92 for the remaining 33 classrooms.

Through the regression analysis for the gravimetrical indoor and outdoor PM_2.5_ mean data of each school, it was estimated that, on average, 53% of the total indoor PM_2.5_ originated from outdoors in the winter months and 47% was generated indoors, with an average F_inf_ of 0.38 (Figure 3).

**Table 3:** Descriptive statistics for the indoor and outdoor 5-d PM concentrations (µg/m^3^) and CO_2 _levels (ppm). AERs (h^−1^) and Q_p_ (L/sp) are also reported. *n* = number of classrooms or outdoor sampling sites in which airborne particles and CO_2_ were monitored, AM = arithmetic mean, SD = standard deviation.

	*N*	AM ± SD	Median	Range		*n*	AM ± SD	Median	Range
PM_0.5 _ind					PM_10 _ind				
SC1	8	13.2 ± 6.5	13.4	4.4–21.7	SC1	8	130.3 ± 55.8	114.4	60.2–219.8
SC2	12	9.7 ± 4.1	9.8	2.5–15.9	SC2	12	136.0 ± 77.2	128.9	40.9–282.3
Total	20	11.1 ± 5.3	10.1	2.1–21.7	Total	20	133.8 ± 67.9	127.3	40.9–282.3
PM_1 _ind					TSPind				
SC1	8	23.7 ± 8.4	27.1	11.4–33.0	SC1	8	235.0 ± 87.3	229.0	101.3–354.9
SC2	12	16.3 ± 4.5	16.9	5.9–23.0	SC2	12	252.3 ± 129.5	243.9	100.6–504.9
Total	20	19.2 ± 7.2	17.5	5.9–33.0	Total	20	245.4 ± 112.2	235.2	100.6–504.9
PM_2.5 _ind					CO_2_ ind				
SC1	20	39.0 ± 8.7	37.9	23.9–59.2	SC1	21	891 ± 174	859	567–1191
SC2	18	26.7 ± 7.1	29.6	10.7–35.5	SC2	18	870 ± 181	806	698–1370
Total	38	33.2 ± 10.0	32.7	10.7–59.2	Total	39	881 ± 175	840	567–1370
PM_2.5 _out					CO_2_ out				
SC1	7	60.5 ± 26.5	66.5	23.1–106.8	SC1	7	505 ± 37	501	448–552
SC2	6	30.9 ± 11.9	31.5	12.0–48.9	SC2	4	479 ± 11	477	469–492
Total	13	46.9 ± 25.4	43.6	12.0–106.8	Total	11	496 ± 32	490	448–552
PM_5 _ind					AERs				
SC1	8	105.5 ± 43.3	94.3	53.1–177.1	SC1	21	0.35 ± 0.14	0.37	0.10–0.60
SC2	12	103.4 ± 56.2	97.9	31.4–206.6	SC2	18	0.39 ± 0.11	0.38	0.24–0.61
Total	20	104.2 ± 50.2	97.9	31.4–206.6	Total	39	0.37 ± 0.12	0.37	0.10–0.61
Q_p_									
SC1	21	3.0 ± 1.6	2.9	0.7–7.7					
SC2	18	3.2 ± 1.0	3.0	1.8–5.6					
Total	39	3.1 ± 1.4	3.0	0.7–7.7					

### 3.2. Indoor PMconcentrations

To better identify and assess the influence of indoor particle sources on the PM mass concentrations, the indoor PM continuous data were divided into three periods: *school hours (S)*, when the rooms were occupied by students during lessons; *other activities (OA)*, with data recorded during gym class and lunch time, after the end of lessons, and in the early morning when no students were present in the classrooms but other activities, such as cleaning, might be performed; and *night (N)*, from 10:00 PM to 6:00 AM, when the rooms were empty and no human activity was performed (Table 4). 

Statistically significant differences were found between the 5-d levels measured in each classroom during *S* and *N* and during *OA* and *N* for TSP, PM_10_, PM_5_ and PM_2.5_ (*p* ≤ 0.001). Conversely, no significant differences were observed for PM_1_ and PM_0.5_ between the same periods (*p* > 0.05).

**Figure 4:**
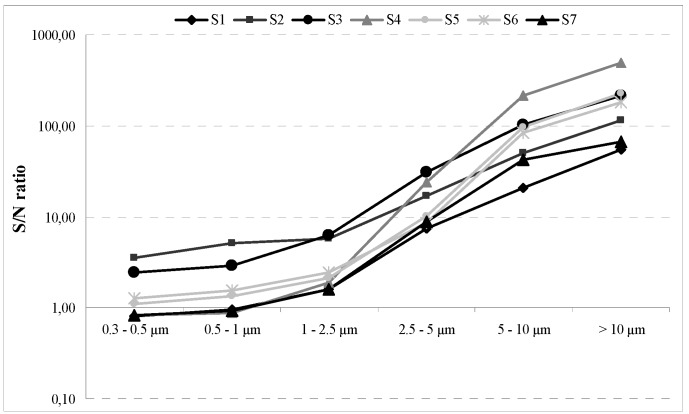
Ratios between the 5-d mean concentrations detected during S and N in the monitored schools. The y-axis is on a logarithmic scale.

Within the range of particle sizes collected by the photometer, the ratios between the 5-d concentrations detected during *S* and *N* were always greater than or close to unity and varied as a function of particle size. The *S/N* values ranged from 0.8 to 5.1 for particle sizes up to 1 µm, then significantly increased (to nearly 500) for coarse particles and particles >10 µm, as depicted in Figure 4.

The indoor PM_10_ was characterized by large variations during *S* and *OA* but exhibited relatively constant levels during *N*. In contrast, the PM_2.5_ variability was low throughout the three different time periods (Table 4). Furthermore, during *S* and *OA* the mean PM_10 _levels were significantly higher than the median values, and markedly higher than the average PM_2.5_ levels, due to the occurrence of extremely high concentrations as a result of the contributions of coarse particles (d.a. = 2.5–10 µm). 

**Figure 5:**
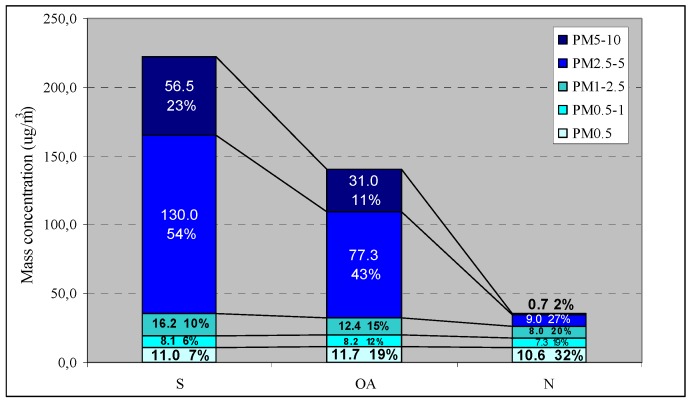
Mean contributions of the size-fractioned PM (PM_0.5_, PM_0.5–1_, PM_1–2.5_, PM_2.5–5_, PM_5–10_) to the 5-d PM_10 _levels. The absolute (µg/m^3^) and relative values (%) are reported in the histograms.

**Table 4:** Descriptive statistics for the 5-d indoor PM (µg/m^3^) and CO_2_ (ppm) concentrations collected during *S*, *OA* and *N* in SC1 and SC2. The PM_2.5_ and CO_2_ I/O ratios for the same periods are also reported; *n* = number of continuous data recorded throughout the sampling sessions, AM = arithmetic mean, SD = standard deviation.

	SC1					SC2				
					I/O ratio					I/O ratio
	*n*	AM ± SD	Median	10th–90th	Mean (range)	*n*	AM ± SD	Median	10th–90th	Mean (range)
PM_0.5_										
*S*	5,499	12.8 ± 8.5	11.7	2.6–24.9		9,148	10.8 ± 8.2	8.0	1.6–19.8	
*OA*	7,328	13.3 ± 8.8	12.9	2.7–26.0		10,964	10.6 ± 9.1	8.4	2.1–21.1	
*N*	5,760	13.6 ± 7.4	12.8	4.7–23.3		9,024	8.8 ± 5.2	8.5	2.3–16.1	
PM_1_										
*S*	5,499	22.9 ± 12.7	23.2	5.5–39.7		9,148	16.9 ± 12.1	14.7	3.2–32.1	
*OA*	7,328	23.2 ± 14.0	24.0	4.8–41.8		10,964	17.8 ± 13.4	14.6	3.9–33.8	
*N*	5,760	23.6 ± 12.0	24.5	7.7–38.9		9,024	14.4 ± 8.0	14.4	3.7–25.8	
PM_2.5 _										
*S*	5,499	41.0 ± 19.7	39.6	15.7–66.1	0.82 (0.47–1.62)	9,148	31.9 ± 22.4	28.5	7.9–57.4	0.85 (0.63–1.28)
*OA*	7,328	37.1 ± 22.4	33.3	9.0–66.2	0.80 (0.49–1.59)	10,964	29.1 ± 23.4	23.2	5.8–54.7	0.90 (0.50–1.39)
*N*	5,760	35.1 ± 22.1	32.7	11.0–61.5	0.71 (0.37–1.35)	9,024	20.1 ± 11.2	19.9	4.8–36.3	0.70 (0.48–0.97)
PM_5_										
*S*	5,499	158.2 ± 114.3	129.2	58.5–299.7		9,148	169.7 ± 190.6	118.9	40.4–325.0	
*OA*	7,328	104.5 ± 142.0	68.8	25.2–212.0		10,964	113.0 ± 236.4	49.0	14.6–234.3	
*N*	5,760	46.3 ± 24.2	41.6	22.9–78.3		9,024	27.7 ± 14.7	27.6	6.1–47.4	
PM_10_										
*S*	5,499	205.7 ± 160.0	162.3	67.7–393.9		9,148	231.6 ± 270.1	157.6	53.1–452.3	
*OA*	7,328	129.0 ± 233.4	73.5	26.3–269.5		10,964	148.5 ± 369.8	53.0	15.8–306.8	
*N*	5,760	47.0 ± 24.4	42.1	23.7–80.8		9,024	28.4 ± 14.9	28.5	6.2–48.4	
TSP										
*S*	5,499	421.0 ± 334.2	330.0	140.1–797.8		9,148	469.2 ± 546.5	320.1	116.3– 922.8	
*OA*	7,328	224.9 ± 728.1	86.9	29.4–454.2		10,964	260.2 ± 912.0	64.4	19.2–522.6	
*N*	5,760	49.3 ± 25.6	43.6	24.8–91.4		9,024	29.7 ± 15.9	29.8	6.9–50.1	
CO_2_										
*S*	16,049	1,423 ± 562	1333	728–2,300	2.84 (1.60–3.90)	13,470	1,428 ± 609	1271	802–2,324	2.67 (2.10–3.30)
*OA*	19,803	733 ± 314	620	474–1,138	1.50 (1.10–1.90)	15,921	686 ± 334	573	455–1,037	1.31 (1.06–1.50)
*N*	16,128	556 ± 98	542	444–688	1.18 (0.90–1.80)	13,645	500 ± 63	490	435–576	1.03 (0.92–1.14)

**Table 5:** Spearman correlation coefficients between the indoor PM fractions during *S*, *OA* and *N*.

	*S*					*OA*					*N*				
	PM_0.5_	PM_1_	PM_2.5_	PM_5_	PM_10_	PM_0.5_	PM_1_	PM_2.5_	PM_5_	PM_10_	PM_0.5_	PM_1_	PM_2.5_	PM_5_	PM_10_
PM_0.5_	1.000					1.000					1.000				
PM_1_	0.932	1.000				0.932	1.000				0.904	1.000			
PM_2.5_	0.743	0.889	1.000			0.777	0.915	1.000			0.779	0.962	1.000		
PM_5_	0.438	0.418	0.587	1.000		0.498	0.566	0.750	1.000		0.798	0.932	0.948	1.000	
PM_10_	0.400	0.373	0.537	0.994	1.000	0.454	0.524	0.712	0.996	1.000	0.788	0.924	0.941	0.999	1.000
TSP	0.270	0.266	0.444	0.919	0.948	0.337	0.420	0.628	0.951	0.973	0.752	0.899	0.928	0.990	0.994

Note: All values significant (*p* < 0.05).

Indeed, during school activities the PM_10_ samples were primarily comprised of PM_2.5–10_, which increased, on average, from 9.7 µg/m^3 ^(29% of PM_10_) during *N* to 186.5 µg/m^3^ (77% of PM_10_) when students were in the classrooms (Figure 5). Coarse particles remained the major fraction during OA as well (54% of PM_10_), with an average concentration of 108.3 µg/m^3^. At night, fine particles were dominant (71% of PM_10_), and all the indoor PM fractions under study were highly correlated (r > 0.75) (Table 5). In addition, the PM_1-2.5_ average levels decreased from S to N (from 16.2 µg/m^3^ to 8.0 µg/m^3^, respectively), while the PM_0.5–1_ and PM_0.5_ mean concentrations remained roughly constant throughout the day (Figure 5).

### 3.3. Indoor and Outdoor CO_2_ Concentrations

As expected, the 5-d indoor CO_2_ concentrations were higher than the corresponding outdoor values, with the 5-d indoor CO_2_ levels between 567 and 1,370 ppm and the ambient concentrations between 448 and 552 ppm. 

**Figure 6:**
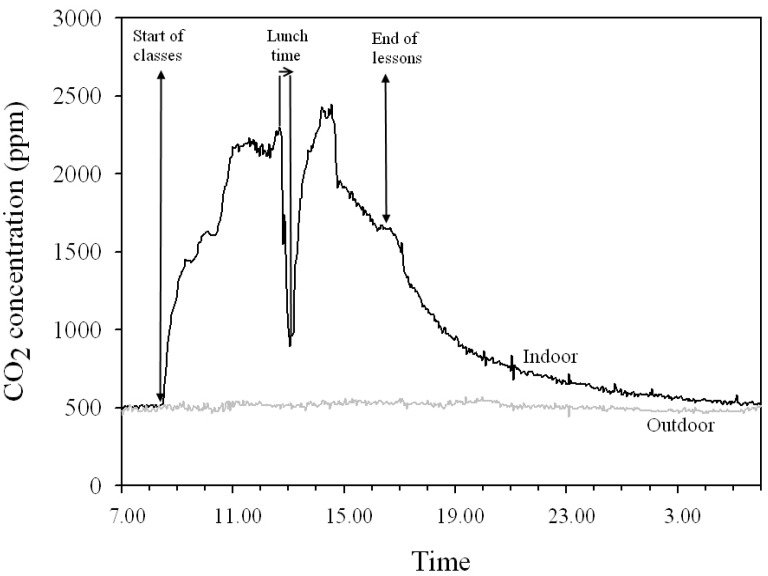
Typical daily indoor variation in CO_2_ levels and the corresponding outdoor trend for one monitored classroom.

The indoor concentrations were highly variable and closely related to the occupation of the rooms (Table 4). A typical diurnal CO_2_ pattern is reported in Figure 6. The decrease of the CO_2_ I/O ratios from, on average, 2.76 during S to 1.11 during N (Table 4) further confirmed this behavior.

AERs estimated with the tracer gas-decay technique (Table 3) represent the worst-case scenario for the ventilation conditions in the studied classrooms because they were calculated when the windows and doors were likely closed (wintertime), and ventilation rates depended only on the air tightness of the building envelope. In naturally ventilated classrooms, as in all studied rooms, the AERs during S should not differ markedly from those estimated in the late afternoon, except when the ventilation increased due to the opening of windows and doors during breaks, lunch time or other periods when the classrooms were unoccupied. No statistically significant correlations were found between the AERs and the average indoor PM concentrations.

Furthermore, the outdoor air supply rates per person (Qp) (Table 3) were estimated for each classroom using the AERs, the class volumes and the average number of students occupying the study rooms according to Persily [19]. The mean value of the estimated rates was close to 3 L/ps.

### 3.4. Comparison of the Pre- and Post-Intervention Indoor PM_2.5_ and CO_2_ Levels

A further aim of the present study was to evaluate the efficacy of some intervention measures (e.g*.*, better cleaning of surfaces with vacuum cleaner and frequent opening of windows) promoted in four schools prior to SC2 as part of a campaign to improve the IAQ in school environments. 

The indoor 5-d PM_2.5_ and CO_2_ concentrations were evaluated for each classroom and paired *t*-tests between the two sampling campaigns were performed for these data sets, separating the classrooms that utilized intervention measures from the classrooms not involved in the intervention program. As regards CO_2_, only data recorded during S were analyzed and the 5-d mean values of each classroom were normalized to the average number of people occupying the room during sampling. Due to the use of different data loggers during SC1 and SC2, the CO_2 _data collected in SC2 were cut to 2,485 ppm to make the two data sets comparable.

The analysis revealed statistically significant differences (*p* < 0.01) between SC1 and SC2 for the classrooms that implemented the recommended intervention measures, with lower post-intervention indoor PM_2.5_ and CO_2_ 5-d concentrations typically observed. In contrast, no statistically significant differences (*p* > 0.05) were found for the classrooms not subjected to the intervention, which were also characterized by higher indoor 5-d CO_2_ levels during SC2 relative to those of SC1 (Table 6). In addition, paired *t*-tests between SC1 and SC2 for PM_2.5_ outdoor 5-d average levels and for PM_2.5_ 5-d mean I/O ratios of the schools involved in the intervention program were performed. The results showed no statistically significant differences neither in PM_2.5_ outdoor data nor in PM_2.5_ I/O ratios. 

**Table 6:** Results of paired *t*-tests between pre- and post- intervention indoor PM_2.5_ and CO_2_ 5-d concentrations.

	PM_2.5_				CO_2_			
	*n*	5-d mean concentrations (µg/m^3^)		*p* (2-tailed)	*n*	5-d mean during S normalized to the average number of students (ppm/p)		*p* (2-tailed)
		SC1	SC2			SC1	SC2	
Rooms with intervention	10	43.1	27.6	0.001	12	73.0	62.3	0.006
Rooms without intervention	6	32.9	27.0	0.150	6	57.6	61.6	0.084
Out (schools subjected to intervention)	4	66.3	30.4	0.164				
		I/O ratio						
Rooms with intervention	10	0.77	0.94	0.286				

### 3.5. Comparison with International Guidelines

Recently, WHO developed specific guidelines for selected indoor pollutants [2], which referred to existing ambient Air Quality Guidelines (AQGs) in the case of indoor PM_2.5_ and PM_10 _[23].

To compare the indoor PM_2.5_ and PM_10_ levels with the 24-hour WHO guidelines (25 µg/m^3^ and 50 µg/m^3^, respectively), we considered for each classroom the average 24-h concentrations for the days in which an entire 24-h sampling was performed (Tuesday, Wednesday and Thursday). Cases in which the monitoring was interrupted due to instrumental or electrical problems were excluded from analysis. 

The indoor PM_2.5_ 24-h levels exceeded the WHO AQG limit on several sampling days (67.9% of the total 24-h indoor concentrations considered).

The WHO AQG levels were also exceeded for the indoor 24-h measurements of PM_10_. Only 8.8% of cases fell below the recommended PM_10_ value.

As regards CO_2_ levels we considered data collected during *S* to better represent the exposure of pupils and teachers to human body odors, noting that the reference guideline value has no specific average exposure times. It was calculated that, on average, for 73% of the time spent by students in the classrooms, the indoor continuous CO_2_ concentrations exceeded the standard recommended by the American Society of Heating Refrigerating and Air-Conditioning Engineers (ASHRAE) of 1,000 ppm [24].

## 4. Discussion

### 4.1. Comparison of PM Concentrations with Historical Data

Overall, the 5-d mean PM_2.5_ and PM_10_ concentrations measured in the present study (Table 3) were quite comparable with the results of previous investigations. A Dutch survey identified average indoor PM_10_ levels between 51 µg/m^3 ^and 166 µg/m^3 ^[25], but was performed in summer, and higher values would likely be expected during winter. A German study examining 54 classrooms in winter and spring found quite low indoor PM_2.5 _concentrations (median = 15 µg/m^3^, range: 5–40 µg/m^3^) [26].

The majority of studies, however, evaluated PM concentrations only during teaching hours when pupils attended class.

The 5-d indoor PM_10_ concentrations during *S* (Table 4) were comparable to the results of Diapouli *et al.* [27], which measured 8-h PM_10_ concentrations of 229 ± 182 µg/m^3^. The respective indoor PM_2.5_ concentrations (82 ± 56 µg/m^3^) were approximately 1.5-fold higher than those measured in our study.

Nevertheless, our 5-d indoor PM_10 _levels during lesson hours were generally elevated in comparison with similar studies. Wheeler *et al.* measured in the U.K. mean PM_10 _winter concentrations of 80 µg/m^3^ [28], while average indoor 8-h PM_10_ levels < 50 µg/m^3 ^were found in Detroit [29]. Noteworthy is that during re-suspension phenomena caused by human presence and activities the re-suspended particles (primarily coarse particles and PM_10 _of crustal origin) can exhibit densities markedly higher than PM_2.5_ (primarily elementary and organic carbon), which was used in our study to correct all the PM optical data. Thus, especially during *S*, the high indoor PM_10_ concentrations registered in the studied classrooms could be overestimated. 

The PM 5-d median values during *S* (Table 4) were comparable to those reported in a German study (118.2 µg/m^3 ^for PM_10 _and 37 µg/m^3^ for PM_2.5_) [12]. Lower median PM_10 _values during teaching hours were found by Janssen *et al.* (81 µg/m^3^) [30] and Fromme *et al.* (92 µg/m^3^) [13].

### 4.2. Indoor and Outdoor Fine PM Concentrations

Contrary to the results of previous studies [12,16], we identified indoor 5-d PM_2.5_ concentrations lower than the associated outdoor levels during both sampling campaigns (Table 3).

During SC1, a lower 5-d outdoor PM_2.5 _was observed only for school S1, primarily because the ambient PM levels were decreased to values close to zero by heavy rains (Table 2). Overall, however, SC1 was characterized by 5-d PM_2.5_ outdoor levels higher than SC2 (Table 3). 

Notably, in the Po Valley the atmospheric stability is high, especially during wintertime, and frequent thermal inversions at low altitude, low mixing layer heights and prolonged foggy periods can occur. Specifically, the sampling during SC1 was affected by a lower rainfall rate and a slightly lower average wind speed in comparison with SC2 (Table 2), thus promoting the accumulation of outdoor pollutants, as occurred for school S4, which exhibited the highest outdoor and indoor 5-d PM_2.5_ levels (106.8 µg/m^3^ and 57.9 µg/m^3^, respectively) found in our survey.

In contrast, the outdoor concentrations in SC2 were approximately 50% lower than those in SC1. The lowest 5-d ambient concentration (12.0 µg/m^3^) was found during a rainy and windy sampling week (S2) (Table 2). In the same week, the lowest indoor 5-d PM_2.5_ concentration (13.5 µg/m^3^) was also registered.

### 4.3. Influence of Indoor Sources on Indoor PM Levels

Although school environments usually lack typical indoor PM sources, such as smoking, wood-burning and cooking, high levels of airborne particles in classrooms are often present nonetheless. 

It has been demonstrated that in indoor microenvironments where there is no specific indoor source of pollution (e.g., combustion processes), human presence and activities may represent the main source of indoor particulates [11,31].

In our work, the highest mean concentrations of PM_2.5_, PM_5_, PM_10 _and TSP were observed when the classrooms were occupied by students (Table 4), whose movements and activities led to the re-suspension of settled particles and also affected indoor PM levels through personal clouds. 

A chemical characterization of the particles collected in our survey was not conducted, but other studies have reported that PM in classrooms is partly of human origin (skin flakes) and partly of mineral origin (building materials such as cement and gypsum as well as chalk and soil material brought in by children’s shoes) [12].

The ratios of the 5-d mean concentrations during *S* and *N* (Figure 4) highlighted the strong influence of occupancy on indoor airborne particle concentrations. The exponential growth of the *S/N* ratios for particles > 1 µm indicated that re-suspension became relevant from particulates of 1 µm in aerodynamic diameter but was predominant for particles in the ranges 2.5–10 µm and > 10 µm, with re-suspension rates increasing with particles size, as demonstrated by other studies [8,15]. Overcrowding of classrooms can contribute to increased re-suspension phenomena. The maximum occupancy in a classroom environment, as recommended by the ASHRAE Standard 62-1989 is 50 people/100 m^2^ [24]. In our study, 23.8% of classrooms exceeded the recommended standard during the first sampling campaign, and three rooms were borderline. During SC2, the Standard was exceeded by 27.7% of the classrooms, with one room lying on the threshold (Table 1).

The high indoor PM levels found during *OA* for particles > 2.5 µm (Table 4) were likely the result of cleaning activities, as the cleaning of rooms was normally performed during this time period. It is well known that cleaning activities, such as dusting or vacuuming, are potentially significant indoor PM sources, especially of coarse-mode particles, causing considerable variations in PM levels over short time periods [32].

In the absence of indoor PM sources, when the classrooms were empty and no activities were being performed, the indoor PM_10_ was primarily composed of PM_2.5 _(Figure 5), as also observed by Branis * et al.* [11]. The influence of human presence and activities on particle levels was also demonstrated by the poor correlation between the fine and coarse PM fractions during *S* and *OA* (Table 5), suggesting that various classroom sources exerted different effects on the indoor particle levels, with the greatest influence on the largest particle sizes. In addition, the higher correlations observed during *N* (Table 5) underlined that, without human activities, no particular indoor source could be identified, and the only relevant determinant of indoor concentrations was the particle infiltration from outdoors. 

In contrast to the results of other studies [11,31], we did not observe a significant correlation between the indoor PM levels, especially for coarse particles and PM_10_, and the occupants’ number and density. This observation could be due to the lack of differences in the number of students among the studied classrooms. Further, the various types and intensity of activities performed in the classrooms by the students (whose details were not available) could determine the high indoor particle levels regardless of the number of occupants.

The estimation of the indoor- and outdoor-generated PM_2.5_ suggested that slightly less than half of the 5-d indoor PM_2.5_ in classrooms originated indoors and 53% infiltrated from outdoors. This results could have arisen because the re-suspension phenomena primarily affected the coarse particle concentrations but also the PM_1-2.5_ fraction, which was found to decrease from *S* to *N* (Figure 5). The slight reduction in the PM_2.5_ I/O ratios when students were not present in the buildings and no activities were being performed (Table 4) confirmed this behavior. Similar results were reported by Crist *et al.*, who demonstrated a decline in PM_2.5_ indoor concentrations and I/O ratios associated with the absence of students in school [33].

As discussed below, the most innovative goal of this study was to evaluate the efficacy of the intervention measures suggested to improve the IAQ in classrooms, since, to date, only a limited number of surveys have tackled the problem of assessing PM exposure following risk mitigation interventions.

### 4.4. Evaluation of the Efficacy of Intervention Measures in School Classrooms

To evaluate the efficacy of the proposed intervention measures, we considered PM_2.5_ and CO_2_ as being possibly and beneficially affected by the improved cleaning of surfaces with vacuum cleaner and/or the frequent opening of doors and windows. No time-activity diaries were provided for obtaining information on windows opening and cleaning activities. Thus, no specific detail regarding the recommended intervention was available.

A comparison for PM_5_, PM_10_ and TSP would be very important because of the considerable influence of cleaning activities on these size particle ranges. Unfortunately, we were unable to consider all of the PM fractions due to the small number of comparable OPC samples between the two monitoring sessions.

Statistically significant differences for PM_2.5_ (*p* = 0.001) were found in the classrooms affected by the intervention measures, with lower post-intervention PM_2.5_ 5-d concentrations. Conversely, no significant differences were found in the classrooms not subjected to the intervention program (*p* = 0.150). Nonetheless, this improvement should not be considered as the direct consequence of risk mitigation interventions, as a parallel decrease in the corresponding outdoor PM_2.5_ levels was registered for the schools involved in the intervention program, although not in a statistically significant way (*p* = 0.164). Furthermore, no statistically significant differences were found between SC1 and SC2 PM_2.5_ I/O ratios (Table 6). We can therefore conclude that the intervention measures proposed to reduce indoor particle levels did not seem to significantly influence the indoor fine PM concentrations. 

Conversely, the statistically significant decrease in the indoor CO_2_ levels reported in SC2 during S (*p* = 0.006) could be attributed to the more frequent opening of doors and windows. No significant differences were found in the classrooms where intervention measures were not implemented (*p* = 0.084). 

These preliminary findings should be further investigated using a larger number of samples and more detailed information about all the possible factors and confounders of the PM and CO_2_ measurements (e.g., climatic conditions and the frequency and intensity of cleaning activities and opening of windows). Furthermore, such an assessment would have greater value if performed using parameters that are significantly influenced by the types of interventions proposed (*i.e.*, coarse particles and PM_10_).

### 4.5. Comparison of Indoor PM and CO_2_ Levels with International Guidelines

The indoor AQGs recently proposed by the WHO aimed to provide a uniform basis for the protection of public health from adverse health effects of exposure to indoor air pollutants [2]. When defining the chemicals to be considered for the development of new indoor guidelines, PM (PM_2.5_ and PM_10_) was included in Group 1, as all the priority pollutants for which the definition of guidelines for indoor air was recommended. Nevertheless, no convincing evidence of a difference in the hazardous nature of particulate matter from indoor sources as compared with those from outdoors was found. Therefore, the PM ambient AQGs defined by the 2005 Global Update were recommended also for indoor spaces [2,23].

In general, the high concentrations that could be achieved in school buildings do not necessarily result in higher health risk to pupils, because the sources and composition of PM in indoor air may differ from those in outdoor air [34]. Among other things, studies highlighted the need of further investigations of the chemical composition and source apportionment of indoor PM, also to better clarify the toxicological and biological relevance of PM exposure in schools in comparison to outdoors [12].

67.9% of the total 24-h PM_2.5_ indoor concentrations found in this study exceeded the WHO AQG limit established to prevent excess mortality and morbidity among human populations.

Furthermore, 40.3% of these cases did not achieve the PM_2.5_ Interim target 3 (37.5 µg/m^3^), implying an estimated increase of 1.2% in short-term mortality; some of the measurements (31%) also exceeded the PM_2.5_ Interim target 2 (50 µg/m^3^), which is related to an estimated 2.5% increase in daily mortality compared with the guideline value [23]. None of the 24-h indoor concentrations exceeded the PM_2.5_ Interim target 1 (75 µg/m^3^), although during SC1 two classrooms in school S4 achieved indoor 24-h PM_2.5_ levels close to that threshold (73.4 µg/m^3 ^and 73.6 µg/m^3^).

PM_10 _is less hazardous to human health because coarse particles are unable to reach the deepest parts of the respiratory tree. In the majority of cases (91.2%), the 24-h WHO guideline value was exceeded, with 78.8% of 24-h levels surpassing the PM_10_ Interim target 3 (75 µg/m^3^). 32.7% of cases exceeding the 24-h limit value also exceeded the PM_10_ Interim target 1 (150 µg/m^3^), with an estimated 5% increase in short-term mortality [23]. 

24-h classroom concentrations were calculated for comparison with the 24-h WHO guidelines in terms of average exposure times, although they are not fully representative of the students’ daily exposure to PM. Pupils, indeed, spent on average of 30% of their week-day time in the school environment, where, moreover, they might have frequented locations and rooms in which the airborne PM exposure could be higher or lower (e.g., gymnasium or library) [27]. Moreover, students passed the remainder of the day in other outdoor or indoor microenvironments—primarily at home—where the indoor PM concentrations were likely to be high [35].

With respect to long-term exposure, the collected data were not directly comparable with the 1-year values recommended for PM_2.5 _and PM_10 _(10 µg/m^3 ^and 20 µg/m^3^, respectively) because our study provided only winter levels. However, an average annual indoor value lower than the mean winter concentration is expected, due to typical PM seasonal trends in European countries [13,17].

According to the ASHRAE Standard 62-1989, indoor CO_2_ levels should not exceed 1,000 ppm to ensure satisfactory comfort [24]. Values exceeding this threshold indicate insufficient fresh air and are associated with a higher frequency of health complaints, primarily due to undesirable levels of body odor, although no health impact on humans has been reported at these concentration levels [22]. Several studies have demonstrated that, during lesson hours, classrooms are often inadequately ventilated, especially in wintertime, with high indoor CO_2_ levels reached [6,13,36].

In our study, the CO_2_ ASRHAE Standard was exceeded for the majority of the time occupants spent in the classrooms (73%). The closing of windows and doors, inadequate ventilation rates and overcrowded rooms could be responsible for the high indoor CO_2_ levels monitored during lessons. Intervention measures that appeared to decrease the indoor CO_2_ levels in SC2 were unable, anyway, to reduce the classroom CO_2_ concentrations below 1,000 ppm during teaching hours (Table 4).

## 5. Conclusions

Indoor and outdoor size-fractioned PM was measured in seven school buildings in Milan, in order to characterize the concentration levels in classrooms, compare the measured concentrations with the recommended guideline values and give a first assessment regarding the efficacy of intervention measures applied to mitigate the exposure to indoor air pollutants.

Results revealed a general situation of poor IAQ in schools and confirmed that PM represents a significant concern in school classrooms, especially for the particle fractions affected by indoor re-suspension phenomena. The presence of important indoor sources of coarse particles (human movements, personal clouds, and cleaning activities) markedly increased PM levels during school hours. PM_2.5_ indoor levels were less affected by indoor sources, with a major impact on the PM_1–2.5_ fraction. Over half of the indoor fine particles were estimated to originate from outdoors. In several cases, indoor PM_2.5_ and PM_10_ 24-h concentrations exceeded the corresponding 24-h guideline values established by the WHO. Furthermore, indoor CO_2_ levels often exceeded the ASHRAE Standard, related with the possible perception of body odors.

Risk mitigation measures are strongly recommended to improve IAQ in classrooms. In general, better cleaning of surfaces can aid the removal of settled particles, preventing their re-suspension, with a major impact on the coarse particle fraction; PM may also be diluted by more adequate ventilation of rooms through the opening of windows or the use of exhaust fans. In addition, increasing the rate of ventilation and decreasing the occupant density could reduce indoor CO_2_ concentrations, with beneficial effects on IAQ and the productivity of the children. 

To a first approximation, the intervention proposed between SC1 and SC2 to reduce indoor particle levels didn’t seem to significantly influence the indoor fine PM concentrations, probably due to the limited available information and the limited number of classrooms involved in the risk mitigation program. The intervention effectiveness would likely be more pronounced if coarse particles or PM_10_ would be selected for study. As regard CO_2_, the more frequent opening of doors and windows seemed to significantly reduce the average CO_2 _indoor levels in naturally ventilated classrooms. 

In light of these preliminary findings and the lack of studies assessing PM exposure following intervention of risk mitigation in classrooms, similar investigations, supported by a greater number of schools and more detailed information about the frequency and intensity of cleaning activities and opening of windows, are suggested. 
